# Representations of palliative care, euthanasia and assisted dying within advocacy declarations

**DOI:** 10.1080/13576275.2019.1567484

**Published:** 2019-02-04

**Authors:** Hamilton Inbadas, José Miguel Carrasco, David Clark

**Affiliations:** a School of Interdisciplinary Studies, University of Glasgow, Dumfries, UK; b Scottish Episcopal Church, Forres, UK; c APLICA Investigación y traslación, Madrid, Spain; d Glasgow End of Life Studies Group, University of Glasgow, Dumfries, UK; e ATLANTES Research Programme, Institute for Culture and Society, Universidad de Navarra, Pamplona, Spain

**Keywords:** Declarations, palliative care, euthanasia, assisted dying, advocacy

## Abstract

It is well known that there are disagreements between the proponents of palliative care and of euthanasia or assisted dying, often with little common ground,shaping the end of life discourse internationally. Advocacy documents or ‘declarations’constitute a significant feature of this discourse. The aim of this study was to explore the content of such declarations and to focus on what they can tell us about palliative care and assisted dying and their dispositions towards one another. 104 declarations were identified and included in the study, covering the period 1974 to 2017. These declarations were analysed following the principles of thematic content analysis. We classified them based on their primary purpose: those with the goal of advocating for palliative care services, education and research were grouped under ‘palliative care declarations’; those with the primary objective of advocating for or against euthanasia/assisted dying were classified as “euthanasia/assisted dying declarations”. Our analysis revealed that the content of the declarations could be broadly categorised into three dimensions: framing, claiming and demanding. We demonstrate that these declarations reveal a struggle over the construction of meanings relating to palliative care and assisted dying and constitute a valuable resource for the analysis of an unfolding debate.

## Background

In most parts of the world and for an extended period the proponents of palliative care, on the one hand, and of euthanasia or assisted dying, on the other, have been locked in disagreement (Clark, 2016). The mid-twentieth century founders of the modern approach to hospice and palliative care were almost universally opposed to euthanasia on both moral and strategic grounds, and this stance has largely continued over time (Materstvedt et al., 2003). First, the palliative care protagonists argue that it is not the role of medicine to intervene in order to end life (Boudreau & Somerville, 2013, 2014). Second, and over time, they maintain that where comprehensive palliative care is available, then requests for an assisted death will be unlikely to occur or can be ameliorated (McCormack, Clifford, & Conroy, 2012). Palliative care activists continue to argue that the problems which lead to assisted dying requests or to calls for the legalisation of euthanasia can usually be dealt with in ways that do not require death to be hastened (Chambaere, Cohen, Bernheim, Vander Stichele, & Deliens, 2016; Radbruch et al., 2015). They promote an emphasis on quality of life in the face of advanced disease and reject the idea of dying on demand. At best they suggest that discussions on the legalisation of assisted dying should be postponed until the world is properly served by palliative care provision.

By contrast, the arguments in favour of assisted dying tend to be based on secular principles that often include a ‘rights’ element. First among these is autonomy, the principle of ‘self-authorship’ of one’s life, and of one’s death (Landry, Foreman, & Kekewich, 2015; Sjöstrand, Helgesson, Eriksson, & Juth, 2013). Second is the more neo-liberal connotation of ‘choice’ within the healthcare system, which should include not only treatment and care options but also the determination of when, how and where we die (Wilkinson, 2015). Beyond these two principles, supporters of assisted dying also argue pragmatically that palliative care cannot be effective in every case and is not a universal solution to all end of life care needs (Hendry et al., 2013; Quill, Back, & Block, 2016). Accordingly, they seek autonomy, choice and the right to end one’s life, and for this to be enshrined in law and codes of medical practice.

The debates arising from the two camps highlight key themes that continue to shape the end of life discourse internationally. The spectrum of issues demonstrates the views of individuals and organisations engaged in or concerned about the development of end of life care either in particular local regions or countries or at the global level. Such views are often expressed through advocacy documents or ‘declarations’, of various kinds, defined as ‘statement(s) of intent or summaries of the desirable situation to which participants intend to work and to which they would like to encourage others to work’ (Help the Hospices, 2005). We argue that such ‘advocacy interventions’ (Inbadas, Zaman, Whitelaw, & Clark, 2016) provide a lens through which we can capture some important elements in the global end of life discourse, and in particular the competing claims and standpoints of differing interest groups.

In two earlier studies, we mapped the emergence of these declarations (Inbadas et al., 2016; Inbadas, Zaman, Whitelaw, & Clark, 2017), showing their increasing incidence, their changing geographic scope, and their emerging importance at the end of life field. Here, we look in more detail at the content of the declarations and focus in particular on what they can tell us about palliative care and assisted dying and their dispositions towards one another. Our specific objectives are to explore within the declarations:

- The descriptions of palliative care and euthanasia/assisted dying.
- The varied claims and demands that are made.
- The actions and responses that are sought.
- The desired outcomes that are envisaged.
- The relationships and comparisons that can be established between them.

## Methods

During the period March 2015 to June 2017 we identified declarations on end of life issues by systematic enquiry on the Google search engine, using the following keywords: palliative care, end of life, euthanasia, assisted dying in combination with declaration, charter, manifesto, resolution and statement. Eighty declarations were identified by this method. Over the same period, searches on the websites of advocacy organisations on end of life issues yielded a further 25 declarations. Seven declarations were identified following social media leads, and through a blog post appeal, two more declarations were noted. Out of the 114 declarations thus identified, we then excluded 11 from the study since they did not contain the characteristics of advocacy documents, as described above. The remaining 104 declarations had been in published over an extended period, from 1974 to 2017, but most were generated in more recent times.

The 104 declarations included in the study were analysed followed the principles of thematic content analysis. This involves coding verbal or textual material with the specific aim of making inferences about the experiences of social groups or the characteristics of a phenomenon (Smith, 1992). The method involves analysing the dominant themes in a dataset identified through a frequency count analysis (Braun & Clarke, 2006; Penney, Snyder, Crooks, & Johnston, 2011; Vaismoradi, Turunen, & Bondas, 2013). Conforming to this approach, all declarations were reviewed to identify themes based on the frequency of their occurrence. We used NVivo for coding patterns and themes, identifying the frequency count of recurrent themes and establishing relationships between them. The analysis of data was monitored by an investigator triangulation process by two researchers (HI, J-M C) to assure quality and validity (Hlady-Rispal & Jouison-Laffitte, 2014).

The 104 declarations were grouped into three categories. Declarations with the primary purpose of promoting palliative care were classified as *palliative care declarations* (*n* = 36). Those with the primary purpose of advocating for or against acceptance of euthanasia/assisted dying were classified as *euthanasia/assisted dying declarations* (*n* = 62). There were six declarations that did not fit either of the two groups but advocated around the significant end of life issues such as ‘treatment of pain’, ‘access to morphine’ and ‘rights of the dying child’.

## Framing, claiming and demanding

Our analysis revealed that the content of the declarations could be categorised into three dimensions: (1)*framing* relates to how palliative care and euthanasia/assisted dying are positioned within a body of discourse, their specific properties and attributes and how these relate to wider contextual factors; (2) *claiming* identifies the specific contributions of palliative care and euthanasia/assisted dying to end of life experience and the provision of care; and (3) *demanding* are those elements which seek specific actions from governments, organisations and the general public for the advancement of palliative care or euthanasia/assisted dying.

Out of the 104 declarations we identified, 81 (78%) were advocating for palliative care and/or explicitly opposed to euthanasia/assisted dying; 14 (13%) represented a neutral position on euthanasia or were without direct reference to palliative care or euthanasia/assisted dying; and only 9 (9%) declarations were in support of euthanasia and/or assisted dying. This has significant bearing therefore on the frequency counts and proportions of material and the arguments explored here.

### Framing

Our analysis of the advocacy texts revealed several ways in which declarations can ‘frame’ an issue, chiefly through the use of definitions, conceptual assertions and statements of clarification (Table 1):

**Table 1.** Framing.

	Palliative care	Euthanasia/assisted dying
Definitions	Palliative care is an approach that improves the quality of life of patients and their families facing the problems associated with life-threatening illness, through the prevention and relief of suffering by means of early identification and impeccable assessment and treatment of pain and other problems, physical, psychosocial and spiritual	The termination of a human life by a physician or paramedic
		An act of bringing about the death of a person at his or her request
		Administration of a lethal agent by another person to a patient for the purpose of relieving the patient’s intolerable and incurable suffering
		Deliberate killing of someone, with or without that person’s consent
Conceptual assertions	Palliative care is a human right	Choosing the manner and time of one’s own death is a fundamental right
	The denial of adequate pain treatment/relief is a violation of human rights	Patients have the right to be served by doctors and institutions that practice only medicine and are not involved in state-sponsored killing
	Multidimensional support and care for children at the end of life as a right	Euthanasia and assisted dying manifest disregard for the value of human life and are contrary to autonomy and to the fundamental prohibition against active killing as a principle of medical practice
	The right to decline medical treatment as a basic right of the patient and should be respected in the context of end of life care decisions	
Statements of clarification	The belief that cancer deaths are unavoidably painful is erroneous	Death caused by unintended consequences of treatment or medication is not euthanasia
	Increasing the dose of morphine does not result in hastening of death	Withholding or withdrawing treatment and allowing the natural process of death to occur is not euthanasia
		Respecting patients’ right to refuse treatment is not euthanasia

#### Definitions

Many declarations used definitions to frame palliative care, euthanasia and assisted dying. Twelve declarations had a definition of palliative care, out of which seven were contained in euthanasia/assisted dying declarations (58%). Conversely, no palliative care declarations included a definition of euthanasia/assisted dying.

While some declarations used the World Health Organization (WHO) definition of palliative care, several others used similar definitions where the language is drawn from the WHO. They refer to palliative care as an approach and a philosophy that improves the quality of life of patients with life-limiting illnesses and families through addressing psychological and spiritual needs and include some notion of holistic care delivered through a multidisciplinary team.

While such unified framing is identifiable in definitions of palliative care, definitions of euthanasia and assisted dying presented a more complex picture. The tone and the choice of words differed considerably. Some declarations use a more instrumental language: ‘the termination of a human life by a physician or paramedic’ (Association for Medical and Therapeutic Self-Determination, n.d.), ‘an act of bringing about the death of a person at his or her request’ (Hospice and Palliative Nurses Association, 2011), ‘administration of a lethal agent by another person to a patient for the purpose of relieving the patient’s intolerable and incurable suffering’ (Association AM, 1994). Others use morally charged words: ‘deliberate killing of someone, with or without that person’s consent’ (Canadian Conference of Catholic Bishops, 2015), ‘Euthanasia occurs when a doctor, not an illness, kills a patient’ (The Catholic Church in Aotearoa New Zealand, 2011). Nevertheless, the tone or the choice of terminology did not in themselves indicate the specific position of the declaration on euthanasia/assisted dying.

The declarations included a variety of definitions, for: euthanasia (*n* = 11), voluntary euthanasia (*n* = 2), physician-assisted suicide (*n* = 6), assisted dying (*n* = 1) and suicide (*n* = 2), and often attempted to articulate the differences between them. Two declarations pointed out that the word ‘euthanasia’ is used in a very different way in the contemporary context when compared to its earlier meaning of a ‘good death’ or ‘easy death’.

#### Conceptual assertions

Human rights arguments were found in the declarations, notably as an instrument through which suffering could be eliminated. Fifteen declarations depicted palliative care as a human right. Others asserted that the denial of adequate pain treatment or relief is a violation of human rights. One declaration saw access to palliative care as a legal obligation. A separate declaration on the ‘the rights of the dying child’ (Benini, Vecchi, & Orzalesi, 2014) outlined the right to multidimensional support and care for children at the end of life. Four declarations advocating for the legalisation of euthanasia demanded that dying in a manner and at a time of an individual’s own choosing must be respected as a fundamental right. However, there were also references to the acceptance of euthanasia potentially compromising the rights of people with mental illnesses, dementia, as well as older people and medical practitioners. One declaration emphasised the ‘right to be served by doctors and institutions that practice only medicine and are not involved in state-sponsored killing’ (Catholic Bishops of Alberta, 2016). Two declarations saw declining medical treatment as a basic right of the patient which should be respected in the context of the end of life care decisions.

This framing of end of life issues within the discourse often drew upon authoritative wider declarations (e.g. the Universal Declaration of Human Rights) and resolutions of key international organisations (e.g. the United Nations Committee on Economic, Social and Cultural Rights’ inclusion of ‘palliation’ as part of the right to health), thereby seeking added legitimacy.

Ethical arguments were also marshalled as a form of support. These included the insistence that euthanasia and assisted dying represent a disregard for the value of human life (*n* = 10), are contrary to the principle of autonomy (*n* = 3), violate a fundamental prohibitions on active killing (*n* = 4) or contravene the principles of medical practice (*n* = 4). Some suggested, more broadly, that there is insufficient justification for euthanasia/assisted dying.

Several declarations demanded a reorientation to the understanding of requests for euthanasia/assisted dying, holding that such requests are pleas for help and therefore require the relief of suffering and not the ending of life. Declarations presenting such views demanded access to palliative care for all and in some cases advocated a public health approach to attain this goal.

#### Statements of clarification

Some declarations included statements seeking to resolve putative ‘myths’ or ‘misunderstandings’. Those on palliative care addressed misunderstandings surrounding pain treatment, for example, that ‘cancer deaths are unavoidably painful’ (The Poznan Declaration, 1998) or that escalating the dose of morphine hastens of death.

There were nine explanatory statements in 12 euthanasia/assisted dying declarations which sought to articulate what euthanasia is not. The list included death caused by the unintended consequences of treatment or medication, withholding treatment and allowing the natural process of death to occur, withdrawing treatment and respecting patients’ rights to refuse treatment. These were each deemed part of the palliative care approach to medical treatment which seeks neither to hasten, nor to otherwise bring about the death of the patient.

These manifestations of ‘framing’ formed a significant part of the 104 declarations on end of life issues, using, in turn, the techniques of definition, conceptualisation and clarification.

### Claiming

The 104 declarations on end of life issues contained several claims about the specific contributions of palliative care, euthanasia and assisted dying to end of life experience, end of life care, and the consequences for society more broadly (Table 2). We were able to divide these into statements about positive and negative contributions.

**Table 2.** Claiming.

	Palliative care	Euthanasia/assisted dying
Positive contributions	Palliative care offers relief from suffering	Assisted dying offers a peaceful and dignified death
	Promotes quality of life at the end of life	
	Draws on the skills of a multidisciplinary team	
	Cost effective or low cost means for relieving suffering and providing care and quality of life for people at the end of life	
	The experiences gained in palliative care are relevant for shaping end of life care in other disciplines that deal with chronic and incurable conditions	
Negative contributions	Complete relief of suffering is not always possible for all people at the end of life	There is potential for the abuse of legalised euthanasia/assisted dying
		Families and relatives may not directly pressurise anyone to ask for the ending of life, but indirect and unconscious pressure may be inescapable
		Older people, chronically infirm, and dependent people will be denied their human value and equality as persons
	Terminal sedation is not different from assisted dying because both involve patients exercising a choice about how they die where the ‘real’ difference is only the time it takes to extinguish life	May create a real sense of guilt, either in patients for choosing to live and receive care/treatment, or in relatives for not being able to ‘end suffering’ by euthanasia
		A ‘right to die’ would rapidly become a ‘duty to die’
		If killing is accepted as a solution for a single problem, then society can identify hundreds of problems for which killing can also be seen as a solution

Thirty-two declarations highlighted the positive contributions that palliative care brings to end of life care. Twenty of these were specifically palliative care declarations and the remainder (*n* = 12) were declarations campaigning *against* the legalisation of assisted dying and promoting palliative care as a viable alternative. The most frequent claims about palliative were that it offers relief from suffering (*n* = 9), promotes quality of life at the end of life (*n* = 8) and draws on the skills of a multidisciplinary team (*n* = 7). While pain control and symptom management were prominent in the stated attributes of palliative care at the end of life, several declarations also referred to psychological, social and spiritual suffering and how these can be ameliorated. Yet, three euthanasia/assisted dying declarations acknowledged that despite considerable advances made in palliative care, complete relief of suffering is not always possible for all people in all circumstances.

Palliative care is also represented as a cost-effective or low-cost form of intervention. Some declarations claim that the experiences gained in palliative care are relevant for shaping the end of life care in other disciplines that deal with chronic and incurable conditions. They describe withholding or withdrawing futile treatment and the use of terminal sedation or palliative sedation as part of a broad spectrum approach to the effective delivery of palliative care.

Declarations in support of legalising euthanasia/assisted dying offered a different approach to terminal sedation. Some (*n* = 3) present the view that terminal sedation is no different from assisted dying as both involve patients electing for choice about how they die and claim that rather than prolonging dying, using terminal sedation may offer a peaceful and dignified death.

The 104 declarations included very few positive claims for euthanasia/assisted dying. Although those in support of legalisation framed their arguments around autonomy, control and dignity, there were not many direct references to the positive contributions of euthanasia or assisted dying at the end of life. However, some did present arguments that autonomy cannot be extended to a right to kill oneself and that it does not provide any dignity, but rather ‘serve[s] to confirm the individual’s falsely devalued sense of self-worth’ (National Council for Hospice and Specialist Palliative Care Services, 1997).

Much declaration content about the effects of euthanasia/assisted dying emphasised the negative societal consequences (19 references in a total of 12 declarations). These expressed concerns about the abuse of legalised euthanasia/assisted dying or the creation of undue pressure on family members as well as compromising the personal position of specific groups of individuals who might feel obliged to justify why they want to continue treatment when the cheaper and more convenient option of dying is readily available. It could also create a sense of guilt in patients for choosing to live and receive care/treatment, and relatives for not being able to ‘end suffering’ by euthanasia.

Several declarations presented a potential situation where a ‘right to die’ would rapidly become a ‘duty to die’ (The Catholic Church in Aotearoa New Zealand, 2011), suggesting serious consequences for health care and the erosion of trust between patients and their doctors and nurses. Some warned that in a society where abuse of disabled and older people is already a considerable problem, legalisation of euthanasia/assisted dying will worsen the situation, particularly in the context of increasing numbers of elderly people in need of care and where the system and budgetary pressures are rising. They also claim that once killing is accepted as a solution for a single problem, then society can identify hundreds of problems for which killing can also be an option.

The claims about what palliative care, euthanasia and assisted dying offer to end of life care revealed various potential consequences. There was much claiming of the positive contributions that palliative care can make. But it was also acknowledged that there may be some cases where palliative care may not provide a complete solution. Claims about euthanasia/assisted dying, by contrast, were mostly negative and highlighted damaging consequences to vulnerable individuals, health-care culture and to society.

### Demanding

The 104 declarations on end of life issues made several demands from varied audiences, each with positive views about their effects, should the demands be implemented (Table 3). These focussed around: education, service development, policy change and resources.

**Table 3.** Demanding.

	Palliative care	Euthanasia/assisted dying
Education	Education and training provision for doctors, nurses and other health professionals	Education provision for health professionals to promote their understanding and clarify attitudes towards euthanasia and assisted suicide
	Increased public awareness about palliative care and end of life care issues	Broader discussions to take place on euthanasia/assisted dying
Service development	Provision of palliative care to be considered as the first priority	Option of hastened death to be made available
	Recognising palliative care as a core component of health systems	
	Facilitate the provision of palliative care services in all settings	Rigorous safeguards so that abuse of the availability of assisted dying can be prevented and that no one will be put under pressure to end their life
Policy change	Development, strengthening and implementation of palliative care policies for the increased provision of palliative care	Legalisation of euthanasia/assisted dying and generation of processes that may facilitate a climate for such legalisation
	Integration of palliative care into the medical management of chronically and terminally ill people	Health professionals’ right over whether or not to participate in euthanasia/assisted dying has to be protected
	Removal of barriers to opioid availability, including simplification of legislation, to ensure availability of and easy access to essential medicines and opioids	
Resources	Funding for developing palliative care, including development and implementation of policies, services, education and training, quality improvement, and the availability of essential medicines	Adequate personnel and financial resources for facilitating discussions about varying views on death and dying matters
	Funding and human resources for palliative care research at national and international levels, with special attention to the lack of palliative care research in developing countries	

#### Palliative care

Among those demanding improved palliative care education, most focussed on professional education for doctors, nurses and other health professionals (*n* = 14). Others sought education for the wider public (*n* = 7). There were demands for palliative care and pain management to be included in all training programmes for doctors, nurses and other health-care professionals at undergraduate and postgraduate levels. There were also demands for the provision of ongoing education and training for clinicians. Some declarations set out specific objectives for palliative care education in a context where assisted dying is legal. Here the specific aims were: dispelling myths surrounding pain relief and about palliative and hospice care among doctors, and encouraging consultation with palliative care colleagues where requests are made for assisted dying. This also extended to demands for training to help clinicians with ‘effective communication skills in caring for patients with life-threatening illnesses who request assisted suicide or euthanasia’ (American Nurses Association, 2013).

The general objective for public education was for sensitisation about the need for palliative care and for freedom from pain. Increased public awareness about palliative care and end of life care issues is believed to improve communication between key stakeholders. Educating the public about palliative care is perceived to equip individuals and groups within society to become more confident about discussing wishes and priorities for the end of life care. While most references to public education within the declarations concerned promoting awareness about end of life issues, choices and creating a culture of openness about death and dying, a few also focussed on training and education for volunteers.

Demand for better access to palliative care came *both* from palliative care declarations (*n* = 12) and euthanasia/assisted dying declarations (*n* = 9), calling on governments to give greater priority to palliative care provision. They urge palliative care communities at national and international levels to mobilise support and persuade governing authorities to take action for recognising palliative care as a core component of health systems, which will facilitate provision in all settings. Some made more specific demands for palliative care access for children or for round the clock access to specialist palliative care. Acknowledging the emergence of legalised assisted dying, one declaration called for access to palliative care for all – ‘including those who have expressed a desire for assistance with suicide’.

Demanding a change to national policies was another significant feature of the declarations (*n* = 16). Most demands were to develop, strengthen and implement palliative care policies to expand provision. There were also demands for policy support for the integration of palliative care in the management of chronically and terminally ill people. Some declarations referred to the need for simplification of opioid legislation and for access to pain relief as an essential service. These demands signified the recognition of the importance of policy backing for effective implementation of palliative care, if it is to reach the largest number of people.

Along with policy calls came the demand for resources. Twelve declarations called on governments to provide funding for the development and implementation of palliative care policies, services, education and training, quality improvement, and the availability of essential medicines. Among these, attaining coverage and equity of provision was an important component. Some declarations also appealed for the allocation of funding and human resources for palliative research, nationally and internationally, drawing attention to the lack of palliative care research in developing countries.

Urging governments to make essential drugs available for symptom management emerged as a significant demand (*n* = 14). This ranged from policy amendments to the elimination of regulatory and legal barriers, thereby facilitating easier and affordable access to controlled medications and other essential drugs for palliative care.

#### Euthanasia/assisted dying

The demands expressed in euthanasia and assisted dying declarations mostly related to legalisation and the processes that might facilitate it. Some insisted that rigorous safeguards must be created to prevent abuse or unnecessary pressure on individuals. Similarly, some made demands for the protection of health professionals’ rights about whether or not to participate in euthanasia/assisted dying, should these be legalised in any given jurisdiction.

Demands were also expressed for better education for health professionals to promote their understanding and clarify attitudes towards euthanasia and assisted suicide. They also sought provisions to ensure respect for advance directives and skills and expertise to support patients’ autonomous decision-making.

There were multiple demands for a wider discussion on the subject of euthanasia or assisted dying. One particular declaration contained a call to hospice staff and board members to engage in open discussion and debate to consider the impact of the issue of assisted dying. Some demanded a consultation process to include the views of everyone within a wider consideration of legalisation. One declaration maintained that ‘any discussion of physician-assisted death must include a broader conversation about end of life care’ (New Democratic Party, 2015).

The declarations highlighted a number of demands for the enhancement of options and care available at the end of life. These related to a broad spectrum of areas from policy, procedural and structural changes, approaches to service provision and drug availability, education and training, allocation of adequate personnel and financial resources and openness for discussions and varying views on death and dying matters.

## Conclusions

We identified a substantial number of declarations on end of life issues. Within these we found techniques of framing, claiming and demanding to be in evidence. Each of these categories had specific sub-components.

‘Framing’ contained the use of definitions, conceptual assertions and statements of clarification. These gave specificity to the matters at hand and enabled them to be seen in a wider context. The language of definitions imparted authority, particularly when they came from or were endorsed by prestigious bodies or related to other larger scale declarations, such as those on human rights. The plurality of definitions in evidence, whether they related to palliative care or euthanasia and assisted dying betokens a measure of pluralism, if not significant confusion. Whilst for palliative care, the definition of the WHO was front and centre, no such globally endorsed definition of euthanasia was identified. Within and around the definitions came efforts to conceptualise specific end of life issues, not least the nature and character of palliative care, as well as the boundaries and practices of assisted dying. This, in turn, indicated some areas of overlap, where conceptual clarity was not always in evidence – for example, on issues like terminal sedation, the withholding and withdrawing of treatment, and the propensity of pain-relieving medications to hasten death. In the same ways, the ‘demands’ found in the declarations could overlap and were not always mutually exclusive between the major positions in support of palliative care and euthanasia/assisted dying. Proponents of the latter rarely questioned the value of the former.

The claims made about palliative care and euthanasia/assisted dying fell into competing categories – those that were broadly positive about the contributions made, and those that were negative. Most of the negative effects fell onto euthanasia and assisted dying, whilst palliative care was much more widely cited for its beneficial effects.

The demands made in the declarations had four elements: education, service development, policy change and resources. For palliative care, this had numerous elements from capacity building, to sustainability – focussed on education, training, service provision and the availability of essential medicines. For euthanasia and assisted dying, a one-dimensional demand tended to predominate – the introduction of frameworks to support legalisation.

Many declarations presented the view that there is no place for euthanasia/assisted dying within palliative care, though some revealed more nuanced approaches. Some saw euthanasia as an important component of the end of life care and argued that ‘excellent palliative care should not exclude the right to choose assisted dying’ (The World Federation of Right to Die Societies, 1998). Others suggested that assisted dying should be considered only after all available options have been explored within palliative care. Some viewed palliative care and assisted dying as opposed, some as potentially alongside each other. Some viewed assisted dying as an aspect of palliative care and others as something to be excluded from it.

We observed the notable absence of ‘family’ as a significant consideration in these advocacy documents. Caring for the family of the patient is often presented as part of the core activity of palliative care. It is claimed that the philosophy of palliative care considers family, along with the patient, as the basic unit of care. However, the end of life care declarations that we analysed revealed that the assertions and claims presented were predominantly constructed around the patient’s needs and concerns, and lacked representation of the family. We also observed that the claims and demands made in relation to euthanasia/assisted dying were founded on the individual person’s human right with little consideration to the family’s collective identity and loyalty. This could be regarded as a reflection of the Western worldview, from where many of these declarations originated. Yet, even those with global geographical scope do not capture the rights, responsibilities of family members and the consequences of euthanasia/assisted dying for them. Accordingly, they pay little attention to representing the values of the collective identity culture often found in countries of the global South.

We see in these declarations what has been referred to as the struggle over the production and mobilisation of meanings (Benford & Snow, 2000). It is an active and dynamic process commonly found in social movements. Within the declarations, we studied there were many examples where complex issues were simplified in ways that might help to galvanise support or alternatively, diminish opposition. The declarations were intended to inspire ideals and also to legitimate action. We have shown that such declarations have become an established part of the end of life landscape, yet they are curiously ignored by social researchers. Here we map and categorise the declarations according to content. Future research would be of value in two areas. First, it could evaluate the effectiveness of declarations in achieving the goals they seek. Second, it could focus on the sites of declaration production, the processes of their formation and the insights this can bring into the unfolding development of end of life care discourse.
